# Selection and validation of reference genes for functional studies in the Calliphoridae family

**DOI:** 10.1093/jis/14.1.2

**Published:** 2014-01-01

**Authors:** Gisele Antoniazzi Cardoso, Cleverson Carlos Matiolli, Ana Maria Lima de Azeredo-Espin, Tatiana Teixeira Torres

**Affiliations:** 1 Centro de Biologia Molecular e Engenharia Genética (CBMEG), Universidade Estadual de Campinas (UNICAMP), Campinas, SP, Brazil; 2 Departamento de Genética, Evolução e Bioagentes, Universidade Estadual de Campinas (UNICAMP), Campinas, SP, Brazil; 3 Departamento de Genética e Biologia Evolutiva, Instituto de Biociências, Universidade de São Paulo (USP), São Paulo, SP, Brazil

**Keywords:** BestKeeper, *Cochliomyia*, *Chrysomya*, gene expression, geNorm, NormFinder

## Abstract

The genera *Cochliomyia* and *Chrysomya* contain both obligate and saprophagous flies, which allows the comparison of different feeding habits between closely related species. Among the different strategies for comparing these habits is the use of qPCR to investigate the expression levels of candidate genes involved in feeding behavior. To ensure an accurate measure of the levels of gene expression, it is necessary to normalize the amount of the target gene with the amount of a reference gene having a stable expression across the compared species. Since there is no universal gene that can be used as a reference in functional studies, candidate genes for qPCR data normalization were selected and validated in three Calliphoridae (Diptera) species, *Cochliomyia hominivorax* Coquerel, *Cochliomyia macellaria* Fabricius, and *Chrysomya albiceps* Wiedemann *.* The expression stability of six genes ( *Actin* , *Gapdh* , *Rp49* , *Rps17,* α *-tubulin* , and *GstD1* ) was evaluated among species within the same life stage and between life stages within each species. The expression levels of *Actin* , *Gapdh* , and *Rp49* were the most stable among the selected genes. These genes can be used as reliable reference genes for functional studies in Calliphoridae using similar experimental settings.

## Introduction

The Calliphoridae family contains flies of economic, veterinary, and sanitary importance. Flies of this family feed on different sources, such as living tissues of a vertebrate host (obligate parasites) or decaying organic matter (saprophagous behavior). These infestations, known as myiasis, are caused by Calliphoridae species in the larval stage ( Zumpt 1965 ). The evolutionary origins of parasitic behavior in Calliphoridae are unknown. However, given the evolutionary history of this family, it probably occurred in at least three independent events ( Stevens and Wallman 2006 ). It has been proposed that this division between feeding behaviors reflects the progressive evolution of parasitism in Calliphoridae ( Stevens 2003 ).

An interesting approach for investigating this biological question is to compare the expression of genes related to feeding behavior in closely related species, correlating their expression levels with the different feeding behaviors. Within Calliphoridae, the genera *Cochliomyia* and *Chrysomya* contain both obligate and saprophagous flies, which allows the comparison of the different feeding habits between closely related species. Due to its high sensitivity for the detection of PCR products with a fluorescence report-er, realtime qPCR has become the gold standard method for measuring mRNA levels ( Wong and Medrano 2005 ; VanGuilder et al. 2008 ) and therefore an appropriate technique to compare expression levels between the different Calliphoridae species.

To ensure an accurate measure of the level of gene expression, it is compulsory to normalize the amount of the target gene with the amount of a reference gene. The normalization step is important to reduce experimental variability caused by factors such as the initial amount of total RNA, the integrity of the RNA, and the efficiency of the reverse transcriptase ( Wong and Medrano 2005 ).

Since there is no universal gene that can be used as reference in functional studies, and as part of an effort to understand the evolution of parasitic behavior in Calliphoridae, 10 candidate genes were selected for qPCR data normalization. The expression stability was evaluated for six genes in three different species of the Calliphoridae (Diptera) family, *Cochliomyia hominivorax* Coquerel (parasite), *Cochliomyia macellaria* Fabricius (saprophagous), and *Chysomya albiceps* Wiedemann (saprophagous). Here, a stable expression level among different Calliphoridae species and between two different life-stages within each species is provided. To our knowledge, this is the first cross-species validation study of reference genes for qPCR experiments in an evolutionary framework. A set of candidate genes (and primers to amplify them) that can be tested in different experimental settings is also provided. These are important resources for functional studies in the Calliphoridae family.

## Materials and Methods

### Fly collection and maintenance

*Cochliomyia macellaria* and *C. albiceps* were collected respectively in Campinas and Sorocaba, both in São Paulo State, Brazil. Adult flies were captured using a hand net and decaying meat or fish as bait. *Cochliomyia hominivorax* larvae were collected directly from wounds of infested animals in cattle breeding farms in Caiapônia, Goiás, Brazil. Larvae of the three species were reared at 30° C ± 5° C. *Cochliomyia hominivorax* larvae were maintained in a medium consisting of fresh ground beef supplemented with blood and water (2:1). *Cochliomyia macellaria* and *C. albiceps* larvae were fed on rats donated by the São Leopoldo Mandic College in Campinas, São Paulo, Brazil. These rats were euthanized with a lethal dose of anesthetic (1 mL of 50% chloral hydrate) before they were received. Mature larvae of the three species were allowed to pupate in sawdust. Adults were maintained in cages (34 x 50 x 26 cm) at 25° C and fed a diet composed of dried milk, sugar, and yeast ferment.

### Reference gene selection and primer design

Candidate reference genes were selected from previous insect studies. These candidates were chosen from functional studies using qPCR, in which they were used as reference genes, or from studies validating the stability of mRNA levels across different samples in which they showed a stable mRNA level. *Actin* was used as an endogenous control in a study comparing mRNA levels of genes involved in host specialization in *Drosophila melanogaster* , *Drosophila sechellia* , and *Drosophila simulans* ( Dworkin and Jones 2009 ) *.* The genes *α-tub, Gapdh, GstD1, Rp49, RpL13A* , and *RpS18* were selected from a validation study in heads of the western honeybee, *Apis mellifera* , with a bacterial challenge ( Scharlaken et al. 2008 ). Finally, the stability of *α-tub, Gapdh, Rp49* , and *Ef1α100E* in qPCR experiments was evaluated in brains of nymphs and adults of the locust *Schistocerca gregaria* ( Van Hiel et al. 2009 ). Two additional housekeeping genes were selected, *RpS17* and *SdhA* . The sequences of the candidate genes were recovered from the Flybase ( www.flybase.org ) and GenBank ( www.ncbi.nlm.nih.gov/genbank ) databases.

The sequences from the transcriptome of *Co* . *hominivorax* ( Carvalho et al. 2010 ) were aligned against the sequences of *D. melanogaster* using the program tblastx ( Altschul et al. 1990 ) to search for possible orthologs of the genes *Actin* , α *-* tub, Ef1α *100E* , *Gapdh* , *GstD1* , *RpL13A, Rp49* , *RpS17* , *RpS18* , and *SdhA* .

The mapped reads were used to perform a global alignment ( Chenna et al. 2003 ) against the sequences of the 12 *Drosophila* species with whole genome sequences using the Clustal W program ( Clark et al. 2007 ). The alignments were used to identify conserved regions, whereas primer pairs for each candidate gene were designed using Primer3 ( Rozen and Skaletsky 2000 ).

### Amplification of the selected genes

A PCR was performed using genomic DNA of five Calliphoridae (Diptera) species, *C. albiceps* , *Chrysomya Megacephala* Fabricius, *Chrysomya putoria* Wiedemann, *Co. hominivorax,* and *Co. macellaria* , to test the primers designed for the selected candidate genes.

PCR amplifications were performed in a GeneAmp PCR system 9700 (Applied Biosystems, Life Technologies, www.lifetechnologies.com ) with a 20 µL final volume. All reactions contained MgCl2 in a final concentration of 2.0 mM, 0.6 mM of primers, dNTPs in a final concentration of 200 µM, and 1 unit of Taq DNA polymerase (Fermentas, www.thermoscientificbio.com/fermentas ) with 5–30 ng of DNA. After an initial denaturing step of 3 min at 94° C, 35 cycles were performed, each consisting of 50 sec at 94° C, 30 sec at 60° C, and 30 sec at 72° C. A final extension was performed at 72° C for 5 min.

### RNA isolation and cDNA synthesis

The main motivation for this study was the investigation of feeding habits in Calliphoridae. The larval stages were initially chosen because the different feeding habits are exhibited during this stage. However, adult females are responsible for choosing the oviposition sites and, consequently, can play a major role in the evolution of feeding behavior (once the eggs hatch, the larvae have to feed on any resource the female has chosen). Hence, larvae and adult female samples were chosen for the evaluation of the reference genes.

Total RNA was extracted from adult females and 3rd instar larvae of the three species from two different generations. Three separate individuals of each generation were used, resulting in a total of six biological replicates. The Trizol reagent (Invitrogen, Life Technologies) was used according to the manufacturer’s protocol to extract total RNA. RNA integrity was confirmed through agarose gel electrophoresis.

All samples were treated with Turbo DNase (Ambion, Life Technologies) to avoid DNA contamination. The Turbo DNase inactiva-tion was performed by heating the samples at 75° C for 10 min. To avoid RNA degradation when heated, EDTA was added to a final concentration of 2.5 mM. The RNA was quantified using the Qubit fluorometer (Invitrogen) with the Qubit RNA assay kit (Invitrogen) according to the manufacturer’s protocol.

PCRs were performed in a 20 µL final volume using 1 µL of each treated sample. *Rp49* primer pairs were used in a final concentration of 0.4 mM and an annealing temperature of 60° C in the same conditions previously described to check if any of the samples were still contaminated with DNA after the Turbo DNase treatment.

The cDNA syntheses were performed using 0.4 µg of total RNA with the First Strand cDNA Synthesis Kit (Fermentas) according to the manufacturer’s protocol. After the cDNA synthesis, all samples were diluted 10 times for the qPCR assays.

### mRNA levels quantification

qPCRs were performed in a 12.5 µL reaction volume following the manufacturer’s instructions for SYBR Green PCR Master Mix (Applied Biosystems). In each reaction, 1.5 µL of the cDNA sample were used and the primers were in a final concentration of 0,4 µM. The qPCRs were run in technical replicates to assess the intra-assay variation on an ABI 7500 PCR Real Time System (Applied Biosystems) using the following cycling conditions: 2 min at 50° C, 10 min at 95° C, and 40 cycles consisting of 15 min at 95° C and 60 sec at 60° C. To check for possible nonspecific amplification and primer-dimer formation, after the 40 cycles, samples were submitted to a dissociation step consisting of an increase in temperature from 60° C to 95° C (increasing 1° C per minute for 35 min) to obtain the dissociation curve. PCR efficiencies were calculated using the equation E = 10 ^1/slope^ . To measure the expression stability of the selected genes, the ∆Ct method ( Vandesompele et al. 2002 ) was used to calculate the calibrated data. A control sample (a *Co. hominivorax* larva sample *)* with *Rp49* primers was used in each qPCR run to account for inter-run variations. The inter-run variation in the Ct of the control sample was used to correct all raw Cts values *.* For each gene, 3-fold serial dilutions (six dilutions) of cDNA samples were used to construct a standard curve from Ct measures against the log of template quantity.

### Gene stability analysis

The software geNorm ( Vandesompele et al. 2002 ), NormFinder ( Andersen et al. 2004 ), and BestKeeper ( Pfaffl et al. 2004 ) were used to establish suitable reference genes for qPCR data normalization. These three programs have different statistical approaches that can be used to measure how stable a gene expression is between distinct conditions or among different species and devel-developmental stages.

## Results

### Selection and amplification of the candidate genes

Ten candidate genes ( Table 1 ) were selected from previous studies in insects: *Apis mellifera* ( Scharlaken et al. 2008 ), *Drosophila melanogaster* ( Dworkin and Jones 2009 ), and *Schistocerca gregaria* ( Van Hiel et al. 2009 ). Using the sequence information from the *Co. hominivorax* transcriptome (Carvalho et al. 2010) it was possible to search for orthologs of these candidate genes and design specific primer pairs for *Co. hominivorax* ( Table 1 ).

**Table 1.**
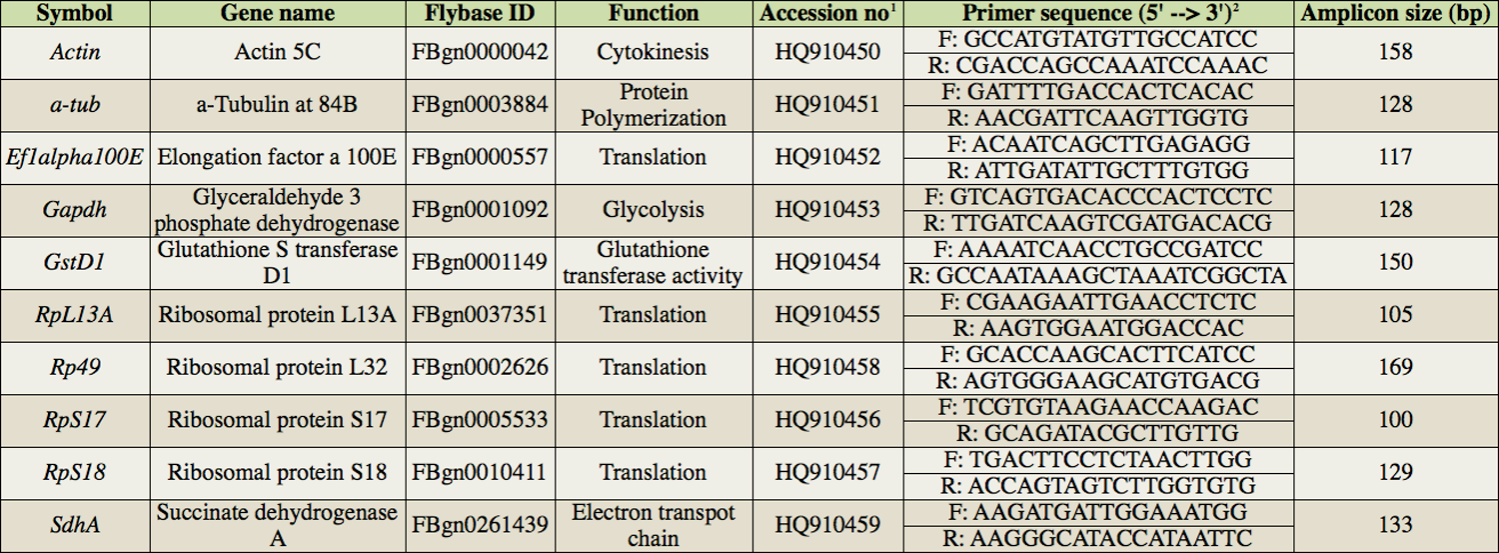
Candidate reference genes.

^1^ Genbank accession number of *Cochliomyia hominivorax* sequence used for primer design

^2^ Primer sequences (F: forward; R: reverse)

The primers were designed using the sequence information from *Co. hominivorax* . Consequently, all primer pairs were specific to this species. To show the utility of the designed primer pairs for a wider range of species, the selected genes were amplified from genomic DNA of five Calliphoridae species ( *C. albiceps* , *C. megacephala, C. putoria, Co* . *hominivorax* , and *Co. macellaria* ). Samples of *C. megacephala* and *C. putoria* were not used in qPCR, as colonies of these species were not maintained and it was not possible to obtain RNA.

The genes *Actin* , *Ef1* α *100E* , *Gapdh* , *GstD1* , *RpL13A* , *Rp49* , *RpS17* , and *RpS18* were amplified from genomic DNA in all five species. *SdhA* was amplified in only two species, *Co. hominivorax* and *Co. macellaria* , while α *-tub* was only amplified in *Co. hominivorax.*

### Quantitative PCR

Gene expression analyses were performed in three species, *C. albiceps* , *Co. hominivorax* and *Co. macellaria.*

The PCR efficiency ranged from 88 to 97% ( Table 2 ). The gene with the lowest efficiency was *GstD1* (88%) in *Co. hominivorax* and *Co. macellaria* . The melting/dissociation curve showed that all qPCRs generated a single specific product. The coefficient of determination, R ^2^ , from standard curves generated for each gene ranged from 0.988 to 0.997, confirming that there were no inhibition contaminants present in the cDNA samples.

**Table 2.**
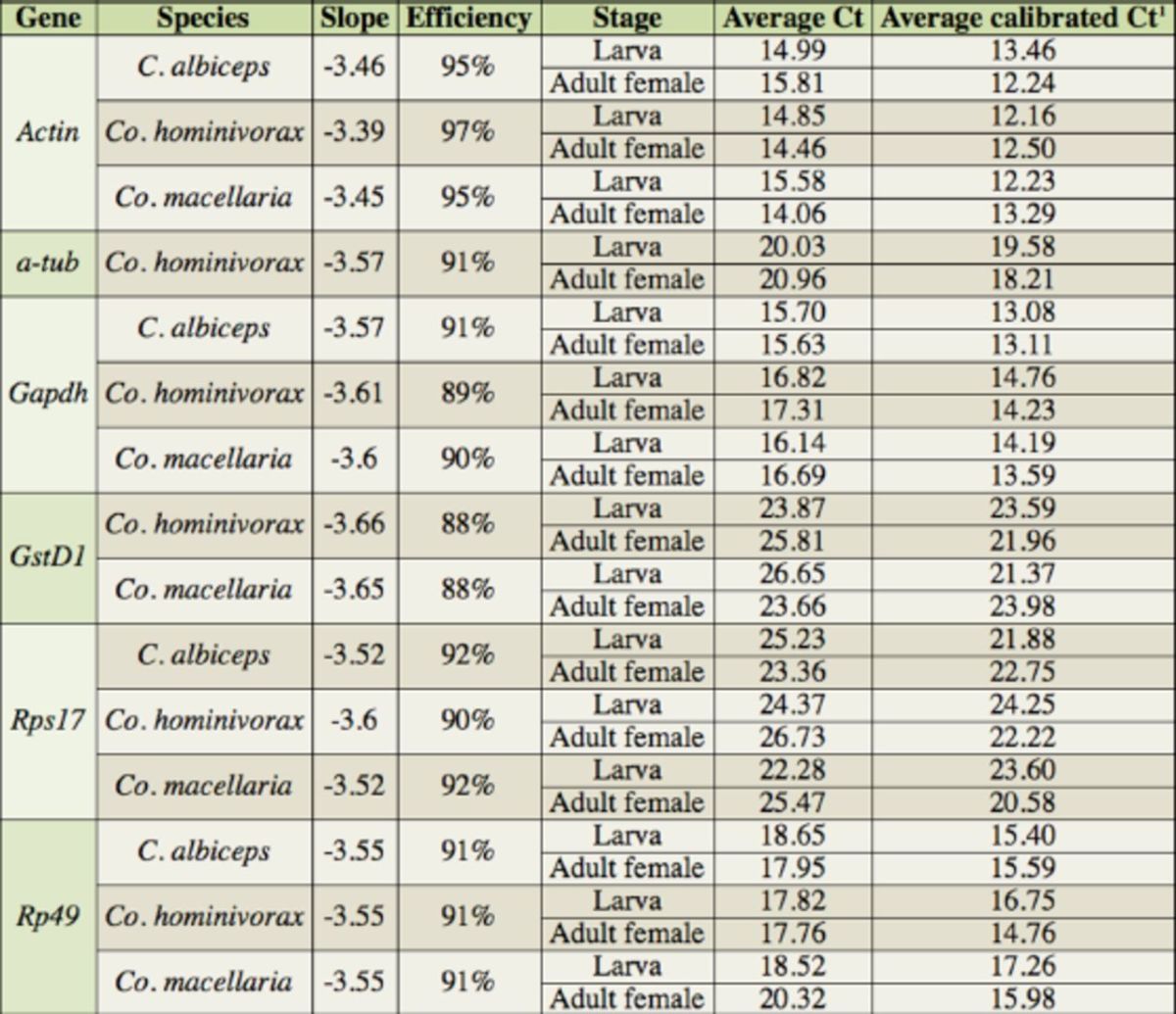
Reaction efficiency and expression of the reference genes.

^1^ A control sample ( *Cochliomyia hominivorax* larva with Rp49 primers) was used in each qPCR run to correct raw Cts for inter-run variation

The expression of *Ef1* α *100E* , *RpL13A* , *RpS18* , and, *SdhA* was not detected in any of the three species, probably due to low mRNA levels. The amplification product for the gene α *-tub* was only detected in *Co. hominivorax* , and *GstD1* had low PCR efficiency in *C. albiceps* . Therefore, the suitable genes for comparing gene expression among the three different species were *Actin* , *Gapdh* , *Rp49* , and *RpS17.*

**Figure 1.**
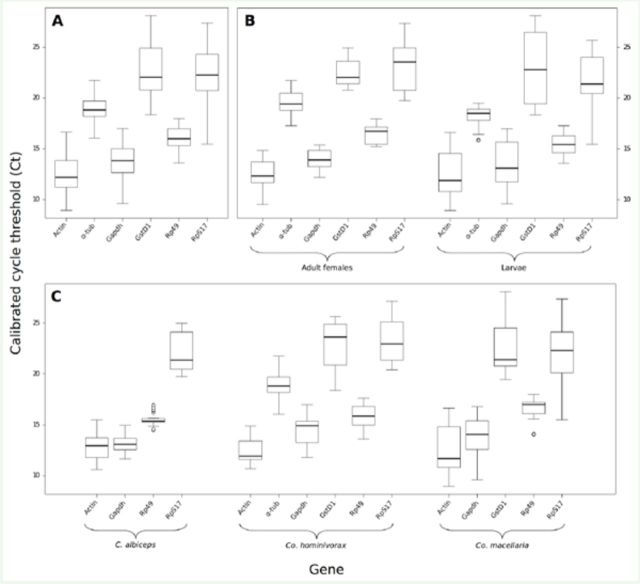
Distribution of the expression levels of the candidate reference genes in different datasets. The distribution of Cts (after inter-run calibration) for each gene is represented using box plots The bold line indicates the median The lower hinge gives the 25% and the upper hinge the 75%. Whiskers (dashed lines) extend to the maximum and minimum sizes. The outliers are marked with circles. (A) Ct distribution in the whole dataset, (B) Ct distribution among species within each life stage and (C) Ct distribution between life stages within species. High quality figures are available online.

The Ct values (calibrated Cts, see Methods) in all samples ranged from 8.94 ( *Actin* in *Co. macellaria* larvae) to 28.09 ( *GstD1* in *Co. macellaria* larvae). The gene with the lowest variation in all dataset was *Rp49* , and the one with the highest variation was *GstD1* ( Figure 1a ). Comparing the Cts within each life stage, *Rp49* had the lowest while *RpS17* the highest variation ( Figure 1b ). Among larval samples, the gene with the lowest variation was *Rp49* and the gene with the high-highest variation was *RpS17* ( Figure 1b ).

Cts between life-stages within species were also compared. In *C. albiceps* and *Co. macellaria* , the gene with the lowest variation was *Rp49* and the highest was *RpS17* ( Figure 1c ). In *Co. hominivorax* , *Rp49* had the lowest variation and *GtsD1* the highest ( Figure 1c ).

The Ct difference between gene-specific replicates was used to calculate the intra-run variation. The mean intra-run variation was 0.21. The variation was higher than 1 (higher than 2-fold) only in the *C. albiceps* sample amplified using primers for the *Rp49* gene, for which the variation was 1.41.

### Comparison among species within the same life stage

The expression stability of the candidate reference genes was evaluated among the different species within each life stage to select reference genes for qPCR experiments for the comparison of different species.

The suitable reference genes for normalization were ranked based on their expression stability (M) in a combined dataset of *Co. hominivorax* , *Co. macellaria* , and *C. albiceps* using geNorm ( Table 3 ). *Gapdh* and *Rp49* were the two most stable genes in samples of adult females.

**Table 3.**
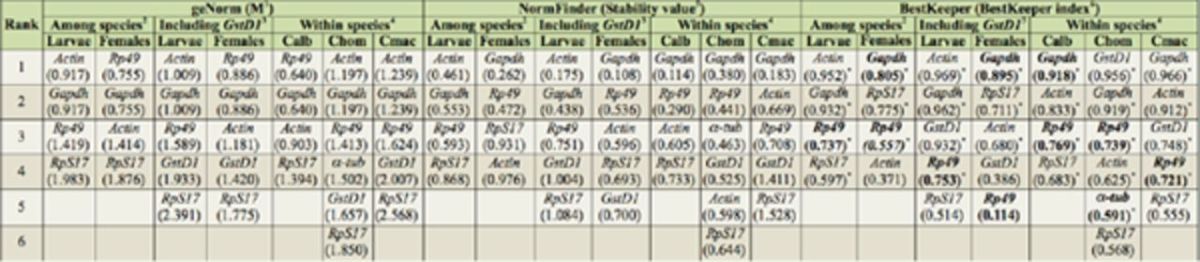
Stability ranking of the reference genes by GeNorm, NormFinder and BestKeeper.

^1^ Gene stability measure as calculated by geNorm.

^2^ Stability of gene expression among the three different species within each life stage

^3^ Stability of gene expression in *Cochliomyia hominivorax* and *Cochliomyia macellaria* including GstD1

^4^ Stability of gene expression between the two life stages within each species

^5^ Stability value as calculated by Normfinder

^6^ Stability index as calculated by Bestkeeper.

*p* < 0.05

Genes in bold had a standard deviation lower than 1

By analyzing the gene expression stability using NormFinder in adult females, a similar result as geNorm was observed ( Table 3 ). The best combination of two genes was *Rp49* and *Gapdh* , with a stability value of 0.332. The software geNorm ranked *RpS17* as the least stable gene, while NormFinder ranked *Actin* as the least stable. Using Bestkeeper, *Gapdh* was the candidate reference gene with the least variation, having an SD of 0.74, which represents a change in gene expression lower than 2-fold (SD smaller than 1 ( Wong and Medrano 2005 )). *Gapdh* was followed by *Rp49* , which had an SD of 0.78. The variation in the expression of the other candidate reference genes was greater than 2-fold. The pairwise correlation between genes and the correlation between each gene and the BestKeeper index was also calculated ( Table 3 ). The best correlation between the BestKeeper index and the candidate reference gene in females was obtained for *Gapdh* (r = 0.805, *p* = 0.001).

In larvae, geNorm ranked *Gapdh* and *Actin* (M = 0.917) as the best reference genes with higher stability values ( Table 3 ). Based on the intra-and inter-group variation in NormFinder, *Actin* (0 *.* 461) and *Gapdh* (0.553) were identified as the most expression-stable, and *RpS17* (0.868) as the least stable.

The analysis of BestKeeper results revealed that only *Rp49* had an acceptable variation in gene expression (SD of 0.84), but it was ranked third according to its correlation with the BestKeeper index ( Table 3 ). Even though the variation in their expression exceeded 2-fold, *Actin* and *Gapdh* had the highest correlation with the BestKeeper index. Hence, there was a consensus among the three different algorithms suggesting the same two genes with the highest expression stability among larval samples of different species.

The results for larvae were similar to those found for adult females. The suitable genes for normalization given by the three programs were *Gapdh* , *Actin* , and *Rp49* .

It was not possible to determine the expression levels of *GstD1* in *C. albiceps* . Hence, the tests were repeated without the *C. albiceps* samples to validate the stability of this gene between *Co. hominivorax* and *Co. macellaria* ( Table 3 ). Regardless of the algorithm, *GstD1* was among the least stable in both larval and adult female samples. Only *RpS17* had a performance worse than *GstD1* .

### Comparison between life stages within species

The stability of the candidate reference genes between larvae and adult females within each species was also tested. Genes with the highest stability values between stages can be used as good reference genes in developmental studies aimed at comparing gene expression among different stages of Calliphoridae flies.

In *C. albiceps* , there was a consensus between NormFinder, geNorm, and BestKeeper results, allowing the identification of two good candidates, *Gapdh* and *Rp49* ( Table 3 ). In BestKeeper, both had a variation lower than 2-fold (SD of 0.42 for *Rp49* and 0.67 for *Gapdh* ). *Actin* was ranked second ( Table 3 ) but it had a higher variation (SD of 1.08). *RpS17* was the least expression-stable gene regardless of the algorithm employed.

In *Co. hominivorax* , *Actin* and *Gapdh* are the best choices based on the geNorm results ( Table 3 ), while NormFinder ranked *Gapdh* and *Rp49* as the most expression-stable genes. BestKeeper ranked the genes differently; *GstD1* and *Gapdh* had the best correlation with the BestKeeper index ( Table 3 ) but only *Rp49* and *Actin* had an acceptable level of variation (SD of 1.01 and 1.05, respectively).

Finally, comparing different stages in *Co. macellaria* , the best reference genes according to geNorm and NormFinder results were *Actin* and *Gapdh* ( Table 3 ). *Gapdh* and *Rp49* had the best correlations with BestKeeper index, but again, only *Rp49* had a variation in gene expression lower than 2-fold (SD of 0.75) *.*

## Discussion

Although qPCR is widely used, there is no consensus as to which gene or gene set should be used for data normalization, and the selection of genes that are expressed in the same levels across all samples and different conditions of a study is still one of the challenges of this technique ( Bustin 2000 ; Bustin 2002 ). Differences in mRNA levels were observed in several housekeeping genes originally considered stable in their expression levels ( Radonic et al. 2004 ; Bustin 2002). Therefore, using non-validated reference genes for normalization may result in an erroneous expression data interpretation. Thus, it is recommended to validate a set of candidate reference genes for each qPCR experiment ( Bustin et al. 2010 ). The validation of reference genes is a difficult task, as the amount of the reference gene itself requires normalization for an accurate measure. An alternative is to use a combined set of candidate reference genes and their pairwise variation in gene expression to evaluate the expression stability of each reference gene ( Kubista et al. 2006 ; Nolan et al. 2006 ).

Recently, with the growing concern over an accurate normalization for qPCR data, there was an increase in the number of validation studies ( Mallona et al. 2010 ). In insect research, there are only a limited number of efforts. Reference genes for qPCR were validated in *A. mellifera* ( Lourenço et al. 2008 ; Scharlaken et al. 2008 ), ticks, *Rhipicephalus appendiculatus* and *Rhipicephalus microplus* ( Nijhof et al. 2009 ), the locust *S. gregaria* ( Van Hiel et al. 2009 ), and the psocid *Liposcelis bostsrychophila* ( Jiang et al., 2010 ). In Diptera, reference genes were validated in the fruit fly *Bactrocera dorsalis* ( Shen et al. 2010 ) and in the blowfly *Lucilia cuprina* ( Bagnall and Kotze 2010 ).

Here, the first effort of a cross-species validation of reference genes for evolutionary studies using qPCR is described. This cross-species approach presents some challenges for the selection of reference genes. First, it is necessary to design primers in conserved regions of orthologous genes. For nonmodel species, as in this study, conserved regions can be found by comparing divergent species. *Cochliomyia hominivorax* sequences were aligned to different species of *Drosophila* . However, not all primers designed recovered the desired product; *SdhA* was amplified only in two species, *Co. hominivorax* and *Co. macellaria* , α *-tub* was amplified only in *Co. hominivorax* , while primers for *GstD1* had a low efficiency of amplification in *C. albiceps* . A second challenge is the selection of genes with a stable expression across the different species. The divergence in the gene expression levels is correlated with the sequence divergence between species ( Castillo-Davis et al. 2004 ; Nuzhdin et al. 2004 ; Lemos et al. 2005 ). For housekeeping genes there is, in general, a higher conservation in both regulatory and coding regions ( Hurst et al. 2002 ; Farre et al. 2007 ). Hence, 10 housekeeping genes commonly used in previous insect studies ( Scharlaken et al. 2008 ; Dworkin and Jones 2009 ; Van Hiel et al. 2009 ) were selected, and primer pairs for these genes were designed based on the sequence information from the *Co. hominivorax* transcriptome ( Carvalho et al. 2010 ).

To evaluate the gene expression stability of the selected genes among different species, the most widely used programs were employed. Each program has its own statistical approach and each calculate a value for the expression stability of each gene. The Excel-based program geNorm ( Vandesompele et al. 2002 ) uses the pairwise variation of every reference gene as the standard deviation of the expression values and calculates a stability value. By using the geNorm algorithm *Gapdh* , *Rp49* , and *Actin* were identified as the most stable candidate genes in this experimental setting.

Similarly, BestKeeper ( Pfaffl et al. 2004 ) calculates an index (BestKeeper index) using the geometric mean of each candidate reference gene. The pairwise correlation is calculated with the BestKeeper index compared to each individual. The genes with best ranking among data in our study were *Actin* and *Gapdh* .

NormFinder ( Andersen et al. 2004) , however, estimates expression stabilities according to the intra-and inter-group expression variation, suggesting the most expression-stable genes. With NormFinder’s algorithm, *Gapdh* , *Rp49* , and *Actin* had the best stability values among the selected genes, both within and among the different species and developmental stages.

Several entomological studies demonstrated that *Actin* , *Rp49* , and *Gapdh* are stably expressed and, therefore, are good reference genes in experiments where normalization is required. Scharlaken et al. 2008 compared the expression stability of six reference genes, *Actin* , α *-tub* , *Gapdh* , *Rpl13A* , *RpS18* , and *Ubq* (UbiQuitin family member; ubq-1), between honeybees with and without a bacterial challenge. *Actin* and *Gapdh* were two of the best reference genes suggested by these authors. *RpL13A* and *RpS18* had higher expression stability values in the honeybee study but the expression of these genes was not detected in our samples.

In the locust *S. gregaria* ( Van Hiel et al. 2009 ), the gene expression stability of nine candidate reference genes in the brain of nymphs and adults were compared. *Actin* , *Ef1* α *100E* , *Gapdh* , and *Rp49* were selected in this study and were also identified as stable reference genes to be used for accurate normalization.

Nijhof et al. 2009 studied the stability of gene expression of nine candidate genes: *Actb* (beta actin), *Btub* (beta tubulin), *Ef1* α *100E, Gapdh* , *GstD1* , *H3F3A* (H3 his-tone family 3A), *PPIA* (Cyclophilin), *RpL4* (Risbosomal protein L4), and *Tbp* (TATA box binding protein) among life-stages of two species, *R. appendiculatus* and *R. microplus* . *Gapdh* was among the most expression-stable genes in the comparison using the combined data of both species. In line with the results of our study, *GstD1* was the least expression-stable gene. However, *Gapdh* did not perform well in all validation studies. In the fruit fly *B. dorsalis* , the expression stability of 10 genes, including *Actin* , α *-tub* , *Gapdh* , and *Ef1* α *100E* , was analyzed in different tissues ( Shen et al. 2010 ). *Actin* performed well as a reference gene, but *Gapdh* did not.

Recently, Bagnall and Koteze (2010) evaluated the stability of reference genes in different life stages of a closely related species in the Calliphoridae family, *L. cuprina.* Three of the 11 genes tested ( *Actin* , *Gapdh* , and *GstD* 1) overlapped with the genes selected in our study. One of the most expression-stable genes in our study, *Gapdh* , was ranked by geNorm and NormFinder as the worst reference gene in the *L. cuprina* study, with M values ranging from 0.121 to 0.643 and NormFinder stability values ranging from 0.195 to 0.724 ( Bagnall and Kotze 2010 ). The authors hypothesized that as *Gapdh* is involved in metabolism, the individuals could have been in different energetic states during sampling. Combined, these results highlight the requirement of validation studies for each experimental setting. Although *Gapdh* performed well in our study and some others, it was also among the least stable genes in some experimental settings.

Commonly used reference genes are involved in essential biological functions, such as cellular transport, translation, structural constitution, and metabolism, because they are thought to have stable expression. However, several studies demonstrated that such housekeeping genes could also vary according to experimental settings ( Huitorel and Pantaloni 1985 ; Chang et al. 1998 ; Vandesompele et al. 2002 ; Axtner and Sommer 2009 ; Nijhof et al. 2009 ; Bagnall and Kotze 2010 ). This expression variation is probably a result of the involvement of these genes in additional cellular functions ( Jain et al. 2006 ).

The genes *RpS17* and *GstD1* were the least stable genes in our study. *RpS17* is involved in translation and encodes a protein related to lipid droplet formation ( Cermelli et al. 2006 ). This lipid component is important for energy storage and utilization ( Arrese and Soulages 2010 ). Furthermore, lipids play important roles in multiple metabolic functions during insect development ( Arrese and Soulages 2010 ). The large variation of the expression of this gene may be related to individual differences in lipid storage between life-stages, but also may reflect different lipid requirements among the studied species. *GstD1* encodes a glutathione-S-transferase involved in detoxification metabolism. This gene is regulated when an insect is exposed to a variety of organic compounds ( Chahine and O'Donnell 2011 ). The compared species have different feeding preferences and were fed in different media. While we did not formally test the differences in gene expression of *GstD1* among the different species, this was an interesting observation and further experiments should be performed to test the association of *GstD1* with feeding habits.

Although several commonly used reference genes displayed a stable expression in many studies, reference genes must be validated for each experimental setting. In our study, the expression stability of six candidate reference genes was tested aiming at future functional studies in the Calliphoridae family. In our analyses, *Actin* , *Rp49* , and *Gapdh* had a stable expression level among different Calliphoridae species and between two different life-stages within each species. Hence, they can be used as reliable reference genes for functional studies in Calliphoridae using similar experimental settings. These reference genes will be critical in studies involving behavior, development, and other biological processes in Calliphoridae species and will be an important resource for other validation studies in Calliphoridae and other insects.
